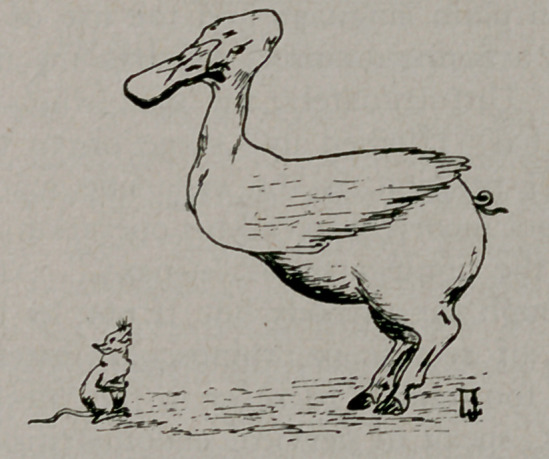# The Ugly Ducklings of the Medical Dialect

**Published:** 1913-09

**Authors:** 


### The Ugly Ducklings of the Medical Dialect

A visitor from Mars would, probably, see nothing more strange in the creature pictured above, than in many others of perfectly normal development. To us, reasonably familiar with terrestrial life, it is plainly a ridiculous impossibility. A zoologist, even in the presence of an actual specimen of such a creature, would be able to point out exactly why it never could have evolved by any conceivable natural process and, could, without hesitation, pronounce it to be the work of a faker.

The drawing, by Dr. Joseph S. Lewis of Buffalo, has been reproduced to point a moral. This peculiar hybrid is exactly comparable to a large number of mongrel words which are being attached to the medical vocabulary. The great majority of the population, having no more knowledge of the evolution of languages than the putative visitor from Mars has of our terrestrial processes of zoologic evolution, regard these words as strange, only because they are encountered for the first time and are impressed with their size and grotesque appearance. Unfortunately, however, there are a good many thousand individuals in this country alone, well enough versed in the languages from which modern medicine is deriving its new terms, to recognize that *duodenitis* (to take one of many hundred possible examples) is just as ridiculous as *speedometer* or *butterine*. A simple homemade tool piece of furniture or word, however rough, can be used without apology. But, when one strives for display and elegance, he must be very sure that what he uses will stand the criticism of the expert whose sneers will eventually penetrate to the ignorant persons who, at first were impressed with the supposed elegance of his affectations.

The use of technical terms is not, in itself, an affectation. As some psychologist has well said, "Words are the nails on which we hang new concepts." If we could not crystalize new concepts into relatively brief words, all progress would be greatly hampered. Never-the-less, it is seldom necessary to apologize for stating a fact in plain language and the use of a technical term borrowed from a foreign source, requires the utmost care as to its correctness. Unfortunately, the man who is most unwilling to say that he has "Hitched up" some organ that has dropped from its normal position, is the very one who fails to realize that while it is not incorrect to call his operation an "—orrhaphy," this term lacks the significance of "—pexy." *Speculum* ought to be technical enough for anybody but, if not, let us speak of proctoscope instead of rectoscope, rhinoscope instead of nasoscope, etc. If we are too modest to refer to the "neck of the womb," we can, at least, speak of cervical endometritis and even accent the first word on the first syllable, or we can say trachelo-metritis —which is not beyond criticism, *but,* rather than call it endocervicitis, let us go to the limit of the speedometer man and call it endoneckitis.

Very recently, there has been formed a league for preserving the purity of the German language against foreign intrusions.

A couple of generations ago the educated Greeks, realizing the decadence of the modern language, set to work systematically to weed out similar intrusions and to restore the language as nearly as possible to its ancient form. So thoroughly has this work been done that a foreigner, with only a moderate familiarity with classic Greek, can read the modern language and can scarcely detect its difference from the ancient. It was stated, as an example, that in a certain newspaper article published within a few months in Athens, there were only three or four words which Xenophen could not have understood, and one of these was "soda water fountain." English is a combination of languages rather than a language. For instance, while by descent, an inflected language, it is almost comparable to the uninflected, monosyllabic Asiatic languages. While theoretically low German, its Saxon words are in a hopeless minority and it has incorporated in recognizable form, almost every native Latin word pretty directly, as well as through the French. It has almost entirely lost the power of forming its own new words and resorts, now, largely to Greek for its technical terms, especially in medicine and allied arts. This being the case, let us take as our motto "Porcus totus aut nullus," and forego the pleasure of coining our own words with such alloys from other languages as suit our individual tastes. There is, indeed, in financial circles, a strong prejudice against private mints, which we might well share. And, since Greek has become, by general consent, the standard of coinage, why not constitute the learned Greeks themselves, the masters of the mint?

## Figures and Tables

**Figure f1:**